# Impact of Sarcopenia and Functional Relationships Between Balance and Gait After Total Hip Arthroplasty

**DOI:** 10.3390/jcm14062036

**Published:** 2025-03-17

**Authors:** So-Yeong Kim, Woon-Su Cho, Chi-Bok Park, Byeong-Geun Kim

**Affiliations:** 1 Department of Physical Therapy, Graduate School, Nambu University, Gwangju 62271, Republic of Korea; 2 Rehabilitation Center, Gwangju 365 Rehabilitation Hospital, Gwangju 62232, Republic of Korea; 3 Department of Physical Therapy, Nambu University, Gwangju 62271, Republic of Korea

**Keywords:** total hip arthroplasty, sarcopenia, balance, gait

## Abstract

**Background/Objectives**: Total hip arthroplasty (THA) is an effective surgical intervention for restoring hip function and alleviating pain caused by osteoarthritis, femoral head avascular necrosis, or fractures. Despite its benefits, postoperative recovery is influenced by various factors, among which sarcopenia plays a critical role. This study aimed to analyze the characteristics of sarcopenia in THA patients admitted to a convalescent rehabilitation hospital and examine its relationship with functional variables such as balance and gait independence. **Methods**: This cross-sectional study included 84 THA patients, categorized into sarcopenia and non-sarcopenia groups using the Asian Working Group for Sarcopenia 2019 criteria. Data were collected on demographic characteristics (e.g., age, gender, height, weight, range of motion (ROM), manual muscle test (MMT)) and functional variables, including balance (Berg Balance Scale, BBS) and gait independence (Functional Ambulation Category, FAC). **Results**: The prevalence of sarcopenia among THA patients was 44.05%. Significant differences were observed between the sarcopenia and non-sarcopenia groups in sex, age, height, weight, ROM, MMT, BBS, and FAC (*p* < 0.05). Logistic regression analysis showed that advanced age increased the likelihood of sarcopenia (OR: 1.072, *p* < 0.05), whereas higher body weight reduced it (OR: 0.784, *p* < 0.05). However, sarcopenia was not significantly associated with balance (BBS: *p* = 0.710) or gait independence (FAC: *p* = 0.990). Instead, a significant positive correlation was found between FAC and BBS (OR: 0.413, *p* < 0.001), as well as BBS and FAC (OR: 0.467, *p* < 0.001), indicating that better balance was associated with greater gait independence and vice versa. Additionally, patients who underwent THA due to fractures had a lower FAC compared to those with osteoarthritis (OR: −0.276, *p* = 0.018). **Conclusions**: Sarcopenia is prevalent among THA patients, and functional variables such as balance and gait independence are closely associated. Additionally, age and body weight were identified as key factors related to sarcopenia. These findings emphasize the importance of early detection and management of sarcopenia in rehabilitation hospital settings and highlight the significance of functional variables in recovery.

## 1. Introduction

Total hip arthroplasty (THA) is an effective surgical procedure performed to restore hip function and alleviate pain. It serves as a treatment method for replacing damaged hip joints caused by various conditions, such as osteoarthritis, avascular necrosis of the femoral head, and fractures [1,2]. This procedure significantly improves patients’ physical function and quality of life, and its prevalence is expected to continue increasing in the future [3]. However, successful postoperative recovery and long-term functional maintenance depend on multiple factors, among which sarcopenia has been identified as a major limiting factor.

Sarcopenia is characterized by a decline in muscle mass, muscle strength, and physical performance and is commonly observed in older adults and various patient populations [4,5,6,7]. Among the key factors of sarcopenia, muscle mass plays a crucial role. In older adults, reduced muscle mass is closely associated with decreased muscle strength, which in turn contributes to impaired balance and slower gait speed [8,9,10]. This condition can lead to multiple complications, including reduced physical function, an increased risk of falls, and a decline in the ability to perform independent daily activities, all of which may also affect patients undergoing THA [11,12,13]. Recent studies have reported that sarcopenia negatively impacts functional recovery and prognosis across various patient groups, underscoring the importance of strategies to prevent and manage this condition [14,15,16]. Therefore, exercise and nutritional interventions should be considered as key strategies for mitigating the effects of sarcopenia and promoting recovery [17].

The prevalence of sarcopenia in THA patients varies depending on diagnostic criteria and surgical indications, with some studies reporting rates ranging from 23.2% to 37.6% [18,19]. In patients undergoing THA, sarcopenia is closely linked not only to reductions in muscle mass and strength but also to key functional variables, such as balance and gait [20,21]. These functional variables are critical indicators for determining a patient’s ability to maintain independent living and achieve successful rehabilitation. Identifying sarcopenia and its associated characteristics in patients admitted to a convalescent rehabilitation hospital can provide valuable insights for optimizing recovery strategies. Therefore, this study aimed to analyze the characteristics of sarcopenia in THA patients admitted to a convalescent rehabilitation hospital and examine its relationship with functional variables, specifically balance and gait independence. By identifying key factors associated with sarcopenia, this study seeks to emphasize the importance of early detection and management to improve rehabilitation outcomes.

## 2. Materials and Methods

### 2.1. Study Design

This study was a cross-sectional analysis of the relationship between sarcopenia status and functional parameters in patients who had undergone THA. The study was conducted on patients hospitalized in a rehabilitation facility, and prior to its commencement, approval was obtained from the Institutional Review Board (IRB) of Nambu University (IRB: 1041478-2023-HR-033). Data collection was conducted from February 2024 to November 2024. The participants were patients who had undergone THA at other hospitals and were subsequently transferred to the research institution for post-acute rehabilitation. Assessments were conducted after obtaining informed consent from the participants upon hospital admission. All collected data were anonymized, and participant confidentiality was strictly maintained in accordance with ethical guidelines.

### 2.2. Study Participants

Participants were selected based on predefined inclusion and exclusion criteria. The inclusion criteria were as follows: patients who had undergone THA, were receiving rehabilitation treatment, and had provided informed consent to participate in the study. Patients were excluded if they had a neurological disorder, were in an acute inflammatory state that made functional assessment difficult or had cognitive impairments that prevented them from complying with the study procedures. Sarcopenia was diagnosed based on the 2019 criteria of the Asian Working Group for Sarcopenia (AWGS), and participants were categorized into either the sarcopenia or non-sarcopenia group accordingly. In this study, bioelectrical impedance analysis (BIA) was used to assess skeletal muscle mass, as recommended by the AWGS 2019 guidelines [4]. BIA was chosen over dual-energy X-ray absorptiometry due to its greater accessibility, lower cost, and ease of use in clinical settings, particularly in rehabilitation hospitals where routine body composition assessments are required.

### 2.3. Outcome Measures and Variables

In this study, the variables were categorized into general characteristics and functional parameters. General characteristics included age, sex, height, weight, reason for surgery (fracture, osteoarthritis, or avascular necrosis of the femoral head), surgical site (left, right, or bilateral), range of motion (ROM), and manual muscle test (MMT). These data were obtained from the electronic medical record, while the ROM and MMT were assessed using methods adapted for functional evaluation in convalescent rehabilitation patients in Korea.

The ROM was measured using a goniometer for both sides of the body, assessing the shoulder (flexion, extension, abduction, external rotation, and internal rotation), elbow (flexion and extension), wrist (flexion and extension), hip (flexion, extension, and abduction), knee (flexion and extension), and ankle (dorsiflexion and plantarflexion). Assessments were conducted with patients positioned for optimal safety and comfort. The upper extremity (shoulder, elbow, and wrist) and ankle ROM were measured in a sitting position. The hip ROM assessment varied depending on the surgical approach: patients who underwent anterior approach THA did not undergo hip extension assessment due to postoperative restrictions (recorded as 0 points), whereas those who underwent posterolateral approach THA were assessed for hip extension in a side lying position. Knee flexion was measured in a supine position instead of the standard prone position, as some post-surgical patients experienced discomfort and fear in the prone position. The scoring system assigned 2 points for normal motion and 1 point for restricted motion, with a total possible score of 64 points.

The MMT was evaluated for both sides of the body, assessing the shoulder (flexion and abduction), elbow (flexion and extension), wrist (flexion and extension), hip (flexion and extension), knee (flexion and extension), and ankle (dorsiflexion and plantarflexion). Assessments were conducted in the same positions as the ROM measurements to ensure consistency. The upper extremity and ankle MMT were evaluated in a sitting position, while the hip extension MMT was assessed in a side lying position for patients with a posterolateral surgical approach and not assessed for those with an anterior approach. The knee flexion MMT was performed in a supine position to accommodate post-surgical patient comfort. The grading system assigned 6 points for the “Normal” grade and 1 point for the “Zero” grade, with a total possible score of 144 points.

Sarcopenia was diagnosed when both the skeletal muscle mass index (SMI) and grip strength were below the established criteria. The SMI was measured using a BIA device (BWA 2.0, InBody, Seoul, Korea), with a threshold of <7.0 kg/m^2^ for men and <5.7 kg/m^2^ for women, according to the 2019 criteria of the AWGS. The grip strength was assessed using a handgrip dynamometer, recording the maximum grip strength of each hand, with a cut-off value of <28 kg for men and <18 kg for women.

Functional parameters were evaluated based on the balance ability and gait independence. The balance ability was assessed using the Berg Balance Scale (BBS), a widely used tool for assessing balance performance in clinical and rehabilitation settings. The BBS consists of 14 items, each scored from 0 (unable to perform the task) to 4 (able to perform the task independently) [22]. The total possible score was 56, with higher scores indicating better balance ability. The BBS was selected for this study due to its ability to evaluate functional balance tasks relevant to daily activities, making it particularly suitable for assessing THA patients recovering in rehabilitation hospitals. Gait independence was assessed using the Functional Ambulation Category (FAC), a validated tool for classifying gait independence based on the level of assistance required during ambulation [23]. The FAC classifies gait independence into six levels, ranging from 0 to 5, with higher scores indicating greater independence in ambulation. The FAC was chosen because it allows for a simple yet effective classification of ambulation ability, making it an appropriate measure for evaluating gait recovery in THA patients.

### 2.4. Statistical Analysis

The collected data were analyzed using Statistical Package for Social Sciences (SPSS) for Windows (Version 23.0, IBM Corp., Armonk, NY, USA) statistical software. Differences between groups were examined using independent sample *t*-tests and chi-square tests. Multiple regression analysis was performed to assess the impact on functional parameters, while logistic regression analysis was conducted to identify factors influencing the likelihood of sarcopenia. The level of statistical significance was set at *p* < 0.05.

## 3. Results

A total of 84 patients who underwent THA participated in this study, among whom 37 were diagnosed with sarcopenia, resulting in a sarcopenia prevalence rate of 44.05%. Comparisons between the sarcopenia and non-sarcopenia groups revealed significant differences in the sex, reason for surgery, age, height, weight, ROM, MMT, BBS, and FAC (*p* < 0.05) (Table 1). The analysis of the effects of the general and functional characteristics on sarcopenia in THA patients indicated that each additional year of age increased the likelihood of belonging to the sarcopenia group by 1.072 times (*p* < 0.05) (Table 2). In contrast, each 1 Kg increase in body weight reduced the likelihood of being classified in the sarcopenia group by 0.784 times (*p* < 0.05) (Table 2).

The analysis of the impact of the general characteristics on the functional parameters (BBS and FAC) in THA patients showed that higher FAC scores were associated with higher BBS scores (OR: 0.413, *p* < 0.05). Similarly, higher BBS scores were associated with higher FAC scores (OR: 0.467, *p* < 0.05) (Table 3). Regarding the reason for surgery, patients who underwent THA due to fractures had lower FAC scores compared to those with osteoarthritis (OR: −0.276, *p* < 0.05) (Table 3).

## 4. Discussion

The prevalence of sarcopenia in THA patients was 44.05%, which is similar to previous studies on total knee arthroplasty and THA [24,25,26]. This finding suggests that sarcopenia is a common condition in older adults undergoing joint replacement surgery. Given that THA is primarily performed in elderly populations, and sarcopenia is closely related to aging; the observed prevalence aligns with prior research findings [27,28]. Additionally, this study identified significant differences between sarcopenia and non-sarcopenia groups in terms of sex, age, weight, ROM, MMT, BBS, and FAC, indicating that sarcopenia may be associated with impaired functional outcomes in this population.

Given the high prevalence of sarcopenia in this population, it is important to understand its association with functional deficits. Comparisons between the sarcopenia and non-sarcopenia groups revealed significant differences in sex, age, height, weight, ROM, MMT, BBS, and FAC. Furthermore, differences in sex and body weight between the sarcopenia and non-sarcopenia groups are consistent with findings from previous studies. Sarcopenia is more prevalent in women, due to lower baseline muscle mass and hormonal changes associated with aging, which may contribute to greater muscle degradation compared to men [29,30]. Additionally, body weight has been identified as a protective factor against sarcopenia, as individuals with higher body weight generally have greater muscle mass [31]. However, excessive body weight may also contribute to obesity-related sarcopenia, necessitating careful evaluation of body composition rather than relying solely on weight as an indicator [32].

Previous studies have reported similar findings, indicating that sarcopenia is associated with poor functional outcomes in older adults undergoing orthopedic surgery. Sarcopenia can develop at any stage, either before or after surgery. Although this study did not specifically account for this aspect, previous research investigating the impact of sarcopenia on THA outcomes found that patients with sarcopenia exhibited significantly reduced gait ability at discharge and increased reliance on assistive devices [33]. These findings are consistent with the results observed in our study population. One possible explanation for these impairments is that sarcopenia leads to deficits in muscle strength, neuromuscular control, and proprioception, all of which are essential for balance and gait stability. Decreased lower limb strength reduces the ability to generate adequate push-off force during walking, leading to a slower gait speed and impaired dynamic stability [34]. Additionally, sarcopenia is associated with neuromuscular degradation, resulting in delayed motor responses and decreased coordination, which further contribute to balance deficits and a higher fall risk [35,36]. These mechanisms may explain why patients with sarcopenia in this study demonstrated significantly lower BBS and FAC scores compared to non-sarcopenic patients. These results suggest that sarcopenia contributes to impaired post-surgical recovery, further emphasizing the importance of early identification and management.

Logistic regression analysis identified sarcopenia risk factors. Age and body weight were significant predictors of sarcopenia occurrence. The positive association between older age and sarcopenia risk is consistent with the natural aging process, which involves a progressive decline in muscle mass [37]. Meanwhile, the negative association between body weight and sarcopenia risk aligns with previous studies suggesting that increased body weight may be linked to greater muscle mass [31]. However, higher body weight does not necessarily indicate healthy muscle mass gain, as obesity-related sarcopenia, where sarcopenia and obesity coexist, must also be considered, particularly in obese individuals [38]. Therefore, a more comprehensive evaluation is necessary.

Multiple regression analysis demonstrated a significant positive association between balance ability and gait independence in THA patients. This suggests that enhancing balance may contribute to improved gait independence [39]. Consequently, rehabilitation programs should incorporate both gait and balance training rather than focusing solely on ambulation. Additionally, patients who underwent surgery due to fractures had lower FAC scores compared to those with osteoarthritis, indicating that fractures may have a more detrimental impact on postoperative recovery [40]. This may be explained by age-related musculoskeletal deterioration, including reduced bone and muscle integrity. The loss of osteocytes and decreased osteoprogenitor activity impair the bone’s response to mechanical stimuli, while muscle aging is characterized by reduced fiber size, decreased protein synthesis, and increased fatty infiltration, all of which compromise contractile function and may hinder recovery following THA [41]. Previous studies have also reported that THA patients with fractures experience delayed functional recovery compared to those with osteoarthritis [42]. These findings align with our results, further supporting the notion that surgical indication influences postoperative functional outcomes in THA patients.

The findings of this study suggest that while sarcopenia may not have a direct impact on balance and gait independence, it is associated with other physical characteristics, such as age and muscle mass, that influence post-THA recovery. Given these associations, rehabilitation programs for THA patients should prioritize strength training, neuromuscular exercises, and proprioceptive training to enhance functional recovery. Additionally, nutritional interventions, including adequate protein intake and supplementation when necessary, may support muscle preservation and improve overall rehabilitation outcomes. A multidimensional approach incorporating strength training and tailored rehabilitation strategies may help optimize mobility and independence in THA patients, particularly those at risk of sarcopenia.

This study has several limitations. First, as a cross-sectional study conducted in a single medical institution, its ability to establish causal relationships is limited, and the generalizability of the findings may be restricted. To address this limitation, prospective studies that track the long-term interaction between sarcopenia and functional changes across multiple centers are needed. Second, the social, cultural, and healthcare characteristics of the participants may have influenced the results; therefore, multicenter studies including more diverse populations are necessary. Third, while this study focused on key functional indicators such as BBS and FAC, other important clinical variables such as fall risk, quality of life, and activities of daily living were not included. Future research should incorporate these variables to provide a more comprehensive assessment of the impact of sarcopenia on overall patient function and quality of life.

## 5. Conclusions

This study examined the prevalence of sarcopenia and its association with functional outcomes in THA patients. Sarcopenic patients exhibited poorer functional status, with age and body weight identified as key predictors. Additionally, fracture as a surgical indication was linked to lower gait independence, and balance ability was positively associated with gait function. These findings highlight the need for targeted rehabilitation strategies in THA patients with sarcopenia. Future prospective studies should explore the long-term impact of sarcopenia on recovery. Additionally, interventions such as resistance training and nutritional support may help mitigate sarcopenia-related functional decline and should be further investigated.

## Figures and Tables

**Table 1:** Comparison of characteristics between the sarcopenia and non-sarcopenia groups.

Variables	Total	Sarcopenia Group	Non-Sarcopenia Group	*p*
Sex (male/female)	21/63	5/32	16/31	0.031 *
Surgery side (left/right/both)	44/34/6	23/13/1	21/21/5	0.174
Reason for surgery (AVN/FX/OA)	7/47/30	2/30/5	5/17/25	<0.001 *
Age (year)	68.38 ± 14.49	75.30 ± 12.63	62.94 ± 13.61	<0.001 *
Height (cm)	158.46 ± 8.84	155.22 ± 7.68	161.02 ± 8.92	0.002 *
Weight (kg)	57.10 ± 10.75	49.98 ± 7.59	62.71 ± 9.53	<0.001 *
ROM (score)	58.67 ± 4.28	57.46 ± 4.99	59.62 ± 3.40	0.021 *
MMT (score)	129.64 ± 13.92	124.57 ± 18.22	133.64 ± 7.25	0.006 *
BBS (score)	24.11 ± 12.88	18.24 ± 12.38	28.72 ± 11.40	<0.001 *
FAC (score)	1.17 ± 0.67	0.95 ± 0.62	1.34 ± 0.67	0.007 *

*: *p* < 0.05, AVN: avascular necrosis; FX: fractures; OA: osteoarthritis; ROM: range of motion; MMT: manual muscle test; BBS: Berg balance scale; FAC: functional ambulation category.

**Table 2:** Analysis of the effects of THA patient characteristics on sarcopenia.

Variables	B	OR	95%CI	*p*
Male	1.733	5.657	0.374~85.546	0.211
Reason for surgery (AVN: OA)	2.422	11.265	0.779~162.953	0.076
Reason for surgery (FX: OA)	1.490	4.439	0.840~23.467	0.079
Age	0.069	1.072	1.012~1.135	0.017 *
Height	0.018	1.018	0.890~1.165	0.792
Weight	−0.243	0.784	0.679~0.905	0.001 *
ROM	−0.047	0.954	0.753~1.208	0.695
MMT	−0.045	0.956	0.882~1.036	0.273
BBS	−0.013	0.987	0.920~1.059	0.710
FAC	0.010	1.010	0.227~4.496	0.990

*: *p* < 0.05, AVN: avascular necrosis; FX: fractures; OA: osteoarthritis; ROM: range of motion; MMT: manual muscle test; BBS: Berg balance scale; FAC: functional ambulation category.

**Table 3:** Analysis of the association between THA patient characteristics and functional variables.

Variables		B	OR	*p*
BBS	Sarcopenia	−1.186	−0.046	0.705
	Female	1.416	0.048	0.720
	Reason for surgery (AVN: OA)	3.689	0.080	0.402
	Reason for surgery (FX: OA)	1.712	0.066	0.553
	Age	−0.145	−0.163	0.131
	Height	0.010	0.007	0.965
	Weight	0.214	0.179	0.182
	ROM	0.527	0.175	0.058
	MMT	0.147	0.159	0.093
	FAC	7.892	0.413	<0.001 *
FAC	Sarcopenia	0.030	0.022	0.863
	Female	−0.409	−0.265	0.059
	Reason for surgery (AVN: OA)	−0.047	−0.019	0.849
	Reason for surgery (FX: OA)	−0.372	−0.276	0.018^*^
	Age	0.000	−0.008	0.942
	Height	−0.021	−0.280	0.077
	Weight	0.004	0.071	0.621
	ROM	−0.010	−0.063	0.527
	MMT	0.000	0.008	0.936
	BBS	0.024	0.467	<0.001 *

*: *p* < 0.05, AVN: avascular necrosis; FX: fractures; OA: osteoarthritis; ROM: range of motion; MMT: manual muscle test; BBS: Berg balance scale; FAC: functional ambulation category.

## Data Availability

The data can be requested from the corresponding author and will be released on reasonable request.

## Abbreviations

The following abbreviations are used in this manuscript:

THA	Total hip arthroplasty
AVN	Avascular necrosis
FX	Fractures
OA	Osteoarthritis
AWGS	Asian working group for sarcopenia
BIA	Bioelectrical impedance analysis
SMI	Skeletal muscle mass index
ROM	Range of motion
MMT	Manual muscle test
BBS	Berg balance scale
FAC	Functional ambulation category
